# Associations of Visceral Adipose Tissue, Circulating Protein Biomarkers, and Risk of Cardiovascular Diseases: A Mendelian Randomization Analysis

**DOI:** 10.3389/fcell.2022.840866

**Published:** 2022-02-03

**Authors:** Yunying Huang, Yaozhong Liu, Yingxu Ma, Tao Tu, Na Liu, Fan Bai, Yichao Xiao, Chan Liu, Zhengang Hu, Qiuzhen Lin, Mohan Li, Zuodong Ning, Yong Zhou, Xiquan Mao, Qiming Liu

**Affiliations:** ^1^ Department of Cardiovascular Medicine, Second Xiangya Hospital, Central South University, Changsha, China; ^2^ Department of Cardiovascular Surgery, Second Xiangya Hospital, Central South University, Changsha, China; ^3^ Department of International Medicine, Second Xiangya Hospital, Central South University, Changsha, China

**Keywords:** obesity, visceral adipose tissue, cardiovascular disease, mendelian randomization, two-sample MR

## Abstract

**Aim:** To evaluate the genetic associations of visceral adipose tissue (VAT) mass with metabolic risk factors and cardiovascular disease (CVD) endpoints and to construct a network analysis about the underlying mechanism using Mendelian randomization (MR) analysis.

**Methods and Results:** Using summary statistics from genome-wide association studies (GWAS), we conducted the two-sample MR to assess the effects of VAT mass on 10 metabolic risk factors and 53 CVD endpoints. Genetically predicted VAT mass was associated with metabolic risk factors, including triglyceride (odds ratio, OR, 1.263 [95% confidence interval, CI, 1.203–1.326]), high-density lipoprotein cholesterol (OR, 0.719 [95% CI, 0.678–0.763]), type 2 diabetes (OR, 2.397 [95% CI, 1.965–2.923]), fasting glucose (OR, 1.079 [95% CI, 1.046–1.113]), fasting insulin (OR, 1.194 [95% CI, 1.16–1.229]), and insulin resistance (OR, 1.204 [95% CI, 1.16–1.25]). Genetically predicted VAT mass was associated with CVD endpoints, including atrial fibrillation (OR, 1.414 [95% CI, 1.332 = 1.5]), coronary artery disease (OR, 1.573 [95% CI, 1.439 = 1.72]), myocardial infarction (OR, 1.633 [95% CI, 1.484 =1.796]), heart failure (OR, 1.711 [95% CI, 1.599–1.832]), any stroke (OR, 1.29 [1.193–1.394]), ischemic stroke (OR, 1.292 [1.189–1.404]), large artery stroke (OR, 1.483 [1.206–1.823]), cardioembolic stroke (OR, 1.261 [1.096–1.452]), and intracranial aneurysm (OR, 1.475 [1.235–1.762]). In the FinnGen study, the relevance of VAT mass to coronary heart disease, stroke, cardiac arrhythmia, vascular diseases, hypertensive heart disease, and cardiac death was found. In network analysis to identify the underlying mechanism between VAT and CVDs, VAT mass was positively associated with 23 cardiovascular-related proteins (e.g., Leptin, Hepatocyte growth factor, interleukin-16), and inversely with 6 proteins (e.g., Galanin peptides, Endothelial cell-specific molecule 1). These proteins were further associated with 32 CVD outcomes.

**Conclusion:** Mendelian randomization analysis has shown that VAT mass was associated with a wide range of CVD outcomes including coronary heart disease, cardiac arrhythmia, vascular diseases, and stroke. A few circulating proteins may be the mediators between VAT and CVDs.

## Introduction

Obesity is an established risk factor of cardiovascular disease (CVD) (Powell-Wiley et al., 2021). However, some epidemiological studies revealed the protective effect of obesity, classified by body mass index (BMI), on CVD outcomes and questioned the nature of the relationship between obesity and CVDs (Antonopoulos et al., 2016a). This paradoxical phenomenon, also known as the “obesity paradox” or “BMI paradox,” indicates that BMI or other general adiposity measurements cannot assess the actual metabolic status and body fat distribution. Central adiposity, especially visceral adipose tissue (VAT)—adiposity accumulated around internal organs—is expected to reflect nature dysmetabolic state in obesity compared with general adiposity. The volume and quality of VAT assessed by computed tomography (CT) were more closely related to metabolic syndrome components than the subcutaneous compartment, regardless of BMI (Shah et al., 2014; Abraham et al., 2015). The associations between VAT area, measured by bioelectrical impedance analysis (BIA), and metabolic risk factors were observed among obese and non-obese participants (Tatsumi et al., 2017).

VAT can be categorized into abdominal adiposity and intrathoracic adiposity. Intrathoracic fat can be further classified as epicardial adipose tissue (EAT), pericardial adipose tissue (PAT), and perivascular adipose tissue (PVAT) according to anatomic location (Oikonomou and Antoniades, 2019). Given the biological heterogeneity of different depots (Hocking et al., 2010; Gaborit et al., 2015), VAT expansion could be both positively or negatively associated with the cardiovascular system (Akoumianakis and Antoniades, 2017). Additionally, CVDs are influenced by VAT and, in turn, influence VAT, especially the adipose depots that anatomical proximity to the cardiovascular system. Epidemiological studies were unable to determine the causal relationship between VAT expansion and CVD outcomes. Together, the relationships between VAT accumulation and the incidence of CVDs are still unclear.

VAT is energy storage and a dynamic endocrine organ that secretes bioactive factors into circulation (Scheja and Heeren, 2019; Ragino et al., 2020). The secretome of VAT includes cytokines (TNF-α, IL-1beta, IL-8), hormones (leptin, adiponectin), growth factors, and others contributing to the pleiotropic effect of VAT. However, the associations of visceral adiposity with a wide range of protein biomarkers of cardiometabolic diseases have not been identified thoroughly. Expanding knowledge of the role of the VAT expansion in cardiometabolic biomarkers leads to the discovery of novel diagnostic biomarkers and therapeutic targets.

It is challenging for observational studies to demonstrate causality between VAT accumulation and CVDs due to confounding and reverse causation bias. We employed Mendelian randomization (MR) analysis to address these issues. MR analysis uses randomly allocated genetic variants as genetic instruments to determine the causal relationship between an exposure and an outcome of interest (Burgess et al., 2019). In the primary study, we implemented the MR approach to evaluate the genetically causal effect of VAT mass on 10 cardiometabolic traits and 53 cardiovascular disease endpoints. In the secondary study, we involved 90 circulating proteins in a network MR analysis to explore the potential mediators engaged in the associations between VAT mass and CVD outcomes. Here, we presented the most comprehensive assessment of the causal relationship between VAT mass and cardiovascular disease to date.

## Materials and Methods

This study relied on publicly available summary statistics from large-scale GWAS. Ethical approval was obtained for all original studies. The data used in this study were analyzed from 14 June 2021 to 1 October 2021.

### Genetic Instruments for Measuring Visceral Adipose Tissue

The genetic association for VAT mass was retrieved from a large GWAS (Karlsson et al., 2019) for predicting VAT mass in 325,153 white British UKB participants. We selected genome-wide significant single nucleotide polymorphisms (SNPs, *p* < 5 × 10^−8^) and excluded correlated SNPs using the “clump_data” function in “TwosampleMR” packages (Hemani et al., 2018) in R software (linkage disequilibrium [LD] *R* ^2^ = .001, >10,000 kb) and attained 221 independent SNPs as instrument variables for VAT mass (Supplementary Table S1).

### Summary Data for Cardiovascular Disease Outcomes

Our outcome variables include 10 metabolic risk factors and 10 CVD endpoints in the primary analysis, 43 CVD endpoints in the secondary analysis. Detailed sample source information is available in Supplementary Table S2.

For metabolic risk factors, we used publicly available summarized data for genetic associations with high-density lipoprotein cholesterol (HDL-C), low-density lipoprotein cholesterol (LDL-C), total cholesterol (TC), and triglycerides (TG) from the Global Lipids Genetics Consortium (Willer et al., 2013); systolic blood pressure (SBP) and diastolic blood pressure (DBP) from the International Consortium of Blood Pressure (Evangelou et al., 2018); type 2 diabetes (T2D) from the Diabetes Genetics Replication and Meta-analysis Consortium (Morris et al., 2012); fasting glucose, fasting insulin, and insulin resistance from the Meta-Analyses of Glucose and Insulin related traits Consortium (Dupuis et al., 2010). These consortia do not include any participants from the UKB.

For CVD endpoints, we used publicly available summarized data for genetic associations with atrial fibrillation (AF, *n* = 1,030,836 individuals of European ancestry) from Nielsen’s study (Nielsen et al., 2018); coronary artery disease (CAD, *n* = 184,305 individuals of mainly European ancestry) and myocardial infarction (MI, *n* = 171,857 individuals of mainly European ancestry) from the Coronary Artery Disease Genome-wide Replication and Meta-analysis plus The Coronary Artery Disease Genetics Consortium (Nikpay et al., 2015); heart failure (HF, *n* = 977,323 individuals of European ancestry) from the Heart Failure Molecular Epidemiology for Therapeutic Targets Consortium (Shah et al., 2020); any stroke (AS, with ischemic stroke [IS] and its three subtypes including large artery stroke [LAS], small vessel stroke [SVS], and cardioembolic stroke [CES], *n* = 150,765 ∼ 446,696 of European ancestry) from the International Stroke Genetics Consortium (Malik et al., 2018); and intracranial aneurysm (IA) from the Intracranial Aneurysm Working Group (Bakker et al., 2020). These datasets included primarily or only individuals of European descent, and no dataset included participants from the UKB study except AF and HF. To avoid biases in causal estimates introduced by sample overlap, we chose the maximal sample sizes in GWAS while minimizing sample overlap between exposures and outcomes (Burgess et al., 2016). Therefore, we omitted the Neale Lab UKB GWAS of CVD endpoints. Similarly, more recent and larger GWAS datasets exist that include the UKB participants for CAD; therefore, we did not utilize the UKB CAD dataset in the present study.

We then leveraged FinnGen Datafreeze 5 release (publicly available 14 May 2021) (FinnGen, 2020) to expand the number of CVD endpoints and replicate the associations with AF, CAD, MI, HF, and stroke, which we selected from independent consortia. The FinnGen Datafreeze 5 analysis included only European ancestry, with summary data for 2,803 available endpoints. We included 43 CVD endpoints from FinnGen release 5, and the sample size ranged from 117,755 to 218,792.

Finally, we used publicly available summarized data for genetic associations with 90 cardiovascular-related circulating proteins from the Systematic and Combined Analysis of Olink Proteins Consortium (Folkersen et al., 2020), many of which were established prognostic biomarkers or treatment targets, measured by the Olink proximity extension assay (PEA) cardiovascular I (CVD-I) panel in 30,931 individuals across 15 studies.

### Statistical Analysis and Sensitivity Analysis

All analyses were performed using the Two-Sample MR (Hemani et al., 2018) and Mendelian Randomization (Yavorska and Burgess, 2017) packages. Before each MR analysis, if a particular SNP was not present in the outcome dataset, we searched and used the proxy SNP (LD *R* ^2^ ≥ 0.8). Exposure and outcome data were then harmonized to ensure that the effect of an SNP on exposure and the effect of that SNP on the outcome corresponded with the same allele.

In the primary analysis, we analyzed the effect of VAT mass on 10 metabolic risk factors, 10 CVD endpoints from independent consortiums, and 43 CVD endpoints from FinnGen study. We used inverse variance–weighted (IVW) MR as the primary method (Burgess et al., 2013), which provided the highest precision while assuming that all SNPs are valid instrumental variables (Burgess et al., 2017). The IVW method provides an unbiased estimate when no horizontal pleiotropy is present or horizontal pleiotropy is balanced. The horizontal pleiotropy exists when the instrumental variant affects pathways other than those of the exposure, which indicates that the instrument is not independent of the outcome. The unbalanced horizontal pleiotropy would disturb the relationship between exposure and outcome of interest. To account for potential pleiotropy, we applied the MR-Egger analysis, weighted median-based regression method, and contamination mixture method as sensitivity analyses. The MR-Egger analysis has low precision but can correct pleiotropy and provide casual inference, even if all genetic variants have pleiotropic effects (Burgess et al., 2017). The weighted median estimates are almost as precise as IVW estimates but require that at least half of the MR instrument weights on the exposure are valid (Burgess et al., 2017). The contamination mixture method is the latest developed method with the lowest mean squared error across a range of realistic scenarios (Burgess et al., 2020). A consistent effect across all four methods shall guarantee high robustness. The Cochran Q heterogeneity test (Bowden et al., 2019) was used to inspect pleiotropy. The MR-Egger intercept test (Bowden et al., 2017) was used to inspect unbalanced pleiotropy. P values of the Egger intercept test (P_Egger intercept_) > .05 indicate no directional pleiotropy between the exposure and outcome of interest. The Steiger directionality test was used to test the causal direction between the hypothesized exposures and outcomes (Hemani et al., 2017). We further conducted a reverse-MR analysis to investigate whether the 10 metabolic risk factors and 10 CVD endpoints affect VAT mass. The genetics instrument for CVD endpoints was selected using the same method as VAT.

We further conducted a network MR analysis to demonstrate the potential mechanisms underlying the associations between VAT and CVDs. Firstly, we analyzed the causal effect of VAT on 90 circulating proteins. Secondly, we analyzed the causal effect of circulating proteins on the total 63 CVD endpoints. In the MR analysis of circulating proteins to CVDs, if there was only 1 SNP available, the “Wald Ratio” method was applied. Only those pairs have consistent directions with outcomes between the associations of VAT with proteins, proteins with CVDs, and VAT with CVDs (beta1*beta2*beta3 > 0, and P1, P2, P3 < .05) were reserved. Finally, 60 VAT-proteins-CVDs axes were identified.

## Results

### Causal Effect of Visceral Adiposity on Metabolic Risk Factors

**FIGURE 1:**
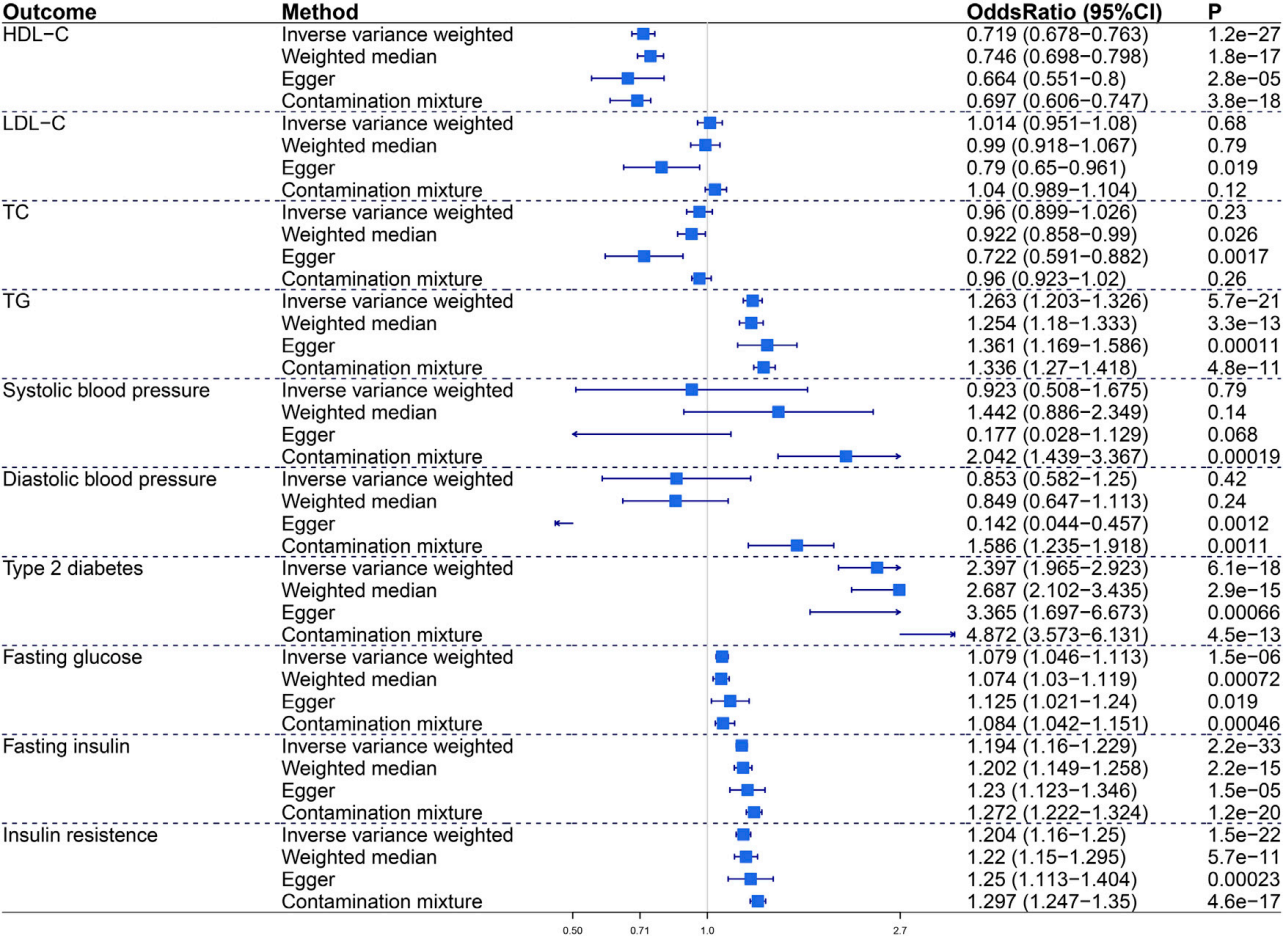
The causal effect of visceral adiposity on 10 metabolic risk factors. Odds ratios are expressed per 1-SD increase in genetically determined VAT mass. CI, confidence interval; HDL-C, high-density lipoprotein cholesterol; LDL-C, low-density lipoprotein cholesterol; TC, total cholesterol; TG, triglycerides.

We assessed the impact of visceral adiposity on metabolic risk factors including lipid profile (HDL-C, LDL-C, TC, TG), blood pressure (SBP, DBP), glycemic traits (fasting glucose, fasting insulin, insulin resistance), and T2D (Figure 1). In primary IVW analysis, genetically predicted increased VAT mass was associated with decreased HDL-C (odds ratio, OR, .719; 95% confidence intercept, CI, .678–0.763; *p* = 1.2e−27), increased TG (OR, 1.263; 95% CI, 1.203–1.326; *p* = 5.7e−21), increased T2D (OR, 2.397; 95% CI, 1.965–2.923; *p* = 6.1e−18), increased fasting glucose (OR, 1.079; 95% CI, 1.046–1.113; *p* = 1.5e−6), increased fasting insulin (OR, 1.194; 95% CI, 1.16–1.229; *p* = 2.2e−33), and increased insulin resistance (OR, 1.204; 95% CI, 1.16–1.25; *p* = 1.5e−22). Sensitivity analyses verified the positive association between VAT mass and metabolic traits. The Egger intercept test indicated no directional pleiotropy in these statistically significant associations.

### Causal Effect of Visceral Adiposity on Cardiovascular Disease Endpoints

**FIGURE 2:**
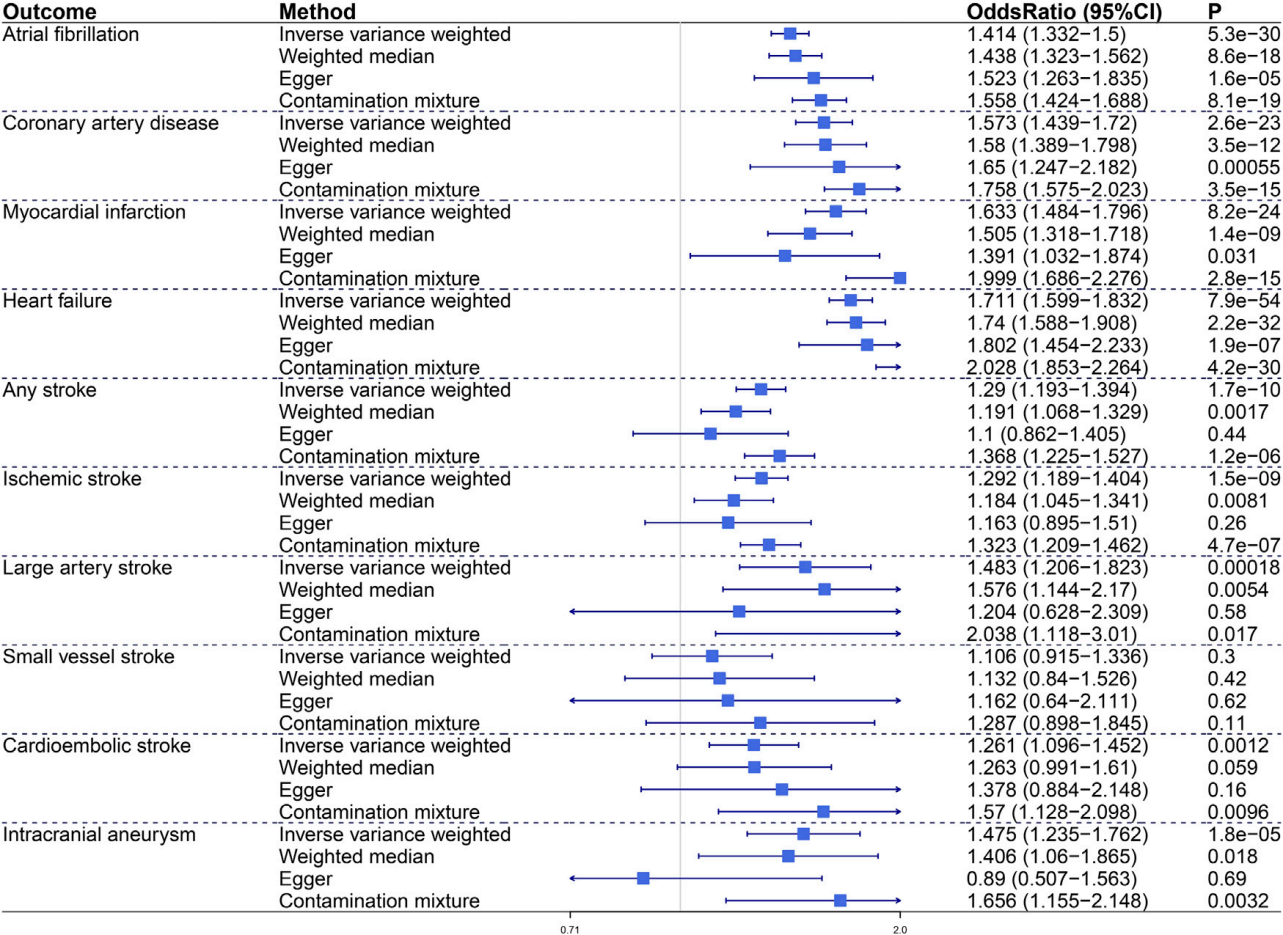
The causal effect of visceral adiposity on 10 independent CVD endpoints. Odds ratios are expressed per 1-SD increase in genetically determined VAT mass. CI, confidence interval.

To characterize the relationship between VAT and CVDs, we conducted MR analyses to investigate the causal effects of VAT on 10 CVD endpoints including AF, CAD, MI, HF, AS, IS, LAS, SVS, CES, and IA (Figure2). Genetic predisposition to VAT expansion increased risks of AF (OR, 1.414; 95% CI, 1.332–1.5; *p* = 5.3e−30), CAD (OR, 1.573; 95% CI, 1.439–1.72; *p* = 2.6e−23), MI (OR, 1.633; 95% CI, 1.484–1.796; *p* = 8.2e−24), and HF (OR, 1.711; 95% CI, 1.599–1.832; *p* = 7.9e−54) in primary IVW analysis. Sensitivity analyses verified the positive association between VAT mass and incident AF, CAD, MI, and HF. Genetically predicted VAT mass presented comparatively small effect on increasing risk of stroke (including AS [OR = 1.29, *p* = 1.7e−10], IS [OR = 1.292, *p* = 1.5e−9], LAS [OR = 1.483, *p* = 0.00018], CES [OR = 1.261, *p* = 0.0012]) and IA (OR, 1.475; 95% CI, 1.235–1.762; *p* = 1.8e−5). Consistent results were obtained in all sensitivity estimate except MR-Egger, which had a wide 95% CI including the null. The Egger intercept test indicated that no directional pleiotropy existed in these relationships.

**FIGURE 3:**
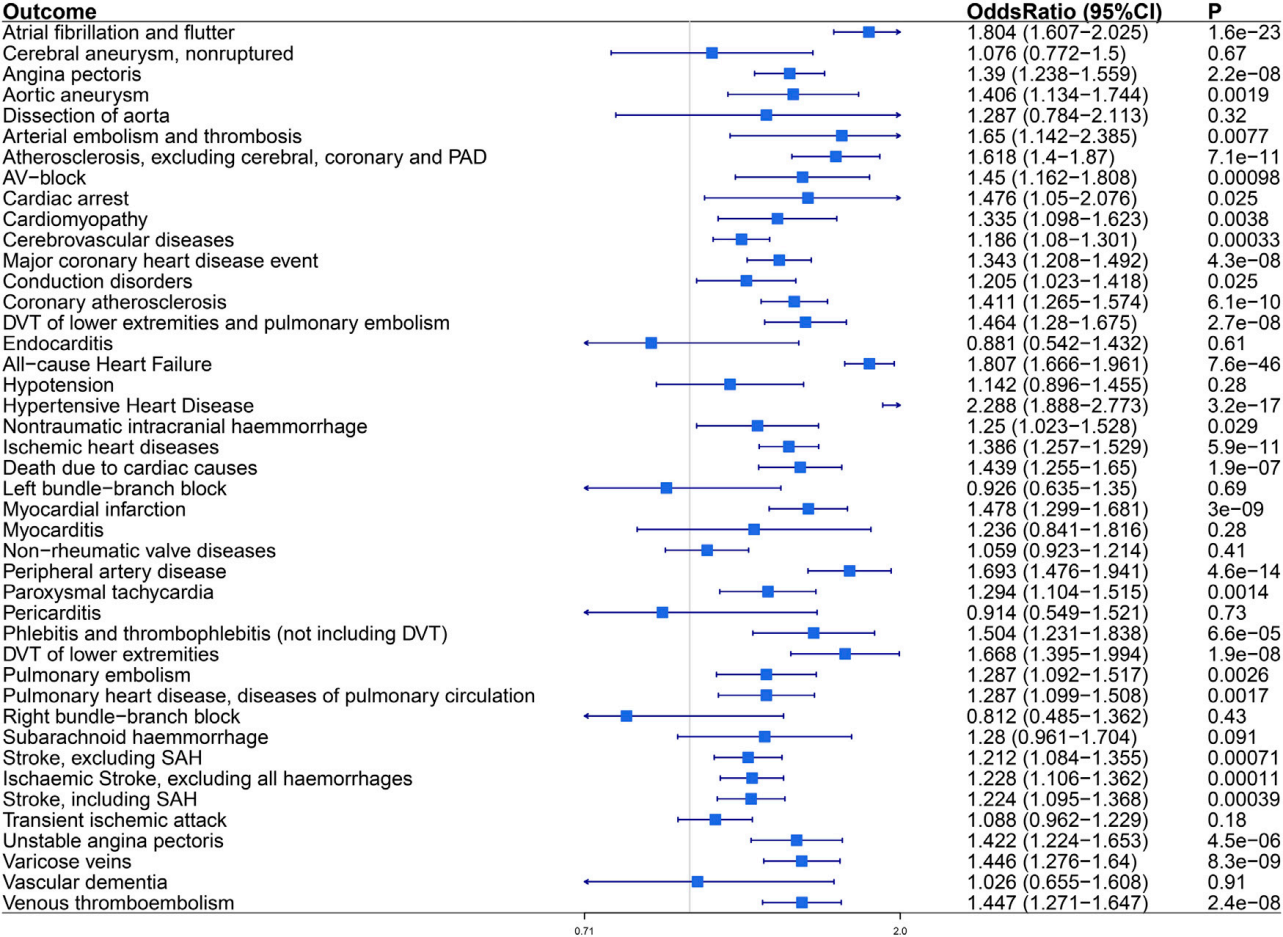
The causal effect of visceral adiposity on 43 CVD endpoints from the FinnGen analysis. Odds ratios are expressed per 1-SD increase in genetically determined VAT mass. CI, confidence interval; PAD, peripheral arterial disease; AV-block, atrioventricular block; DVT, deep vein thrombosis; SAH, subarachnoid haemmorrhage.

In the FinnGen study, the proved VAT–CVDs associations were replicated statistically (Figure 3). The OR (95% CI) per standard deviation (SD) increase in VAT mass was 1.804 (1.607–2.025) for “Atrial fibrillation and flutter,” 1.807 (1.666–1.961) for “All−cause Heart Failure,” and 1.212–1.228 for different subtypes of stroke. For subtypes of coronary heart disease, the OR (95% CI) was 1.39 (1.238–1.559) for “Angina pectoris,” 1.343 (1.208–1.492) for “Major coronary heart disease event,” 1.411 (1.265–1.574) for “Coronary atherosclerosis,” 1.386 (1.257–1.529) for “Ischemic heart disease,” 1.478 (1.299–1.681) for “Myocardial infarction,” and 1.422 (1.224–1.653) for “Unstable angina Pectoris.” Expanding to other CVD endpoints of the FinnGen study, we found the relevance of VAT to hypertensive heart disease, cardiac arrhythmia, vascular diseases, and cardiac death. Genetically determined VAT mass increased risk of “Hypertensive Heart Disease” (OR, 2.288; 95% CI, 1.888–2.773; *p* = 3.2e−17). Besides atrial fibrillation, VAT had impact on other cardiac arrhythmic disease including “AV-block (atrioventricular block)” (OR = 1.45 [1.162–1.808]), “Conduction disorders” (OR = 1.205 [1.023–1.418]), and “Paroxysmal tachycardia” (OR = 1.294 [1.104–1.515]). For diseases of arteries or arterioles including “Aortic aneurysm,” “Arterial embolism and thrombosis,” “Atherosclerosis, excluding cerebral, coronary and PAD (peripheral artery disease),” and “Peripheral artery disease,” the ORs were approximately between 1.406 and 1.693. For diseases of veins including “DVT (deep venous thrombosis) of lower extremities and pulmonary embolism,” “Phlebitis and thrombophlebitis (not including DVT),” “DVT of lower extremities,” “Varicose veins,” and “Venous thromboembolism,” the ORs were approximately between 1.446 and 1.668. Genetically predicted VAT mass increased risk of “Death due to cardiac causes” (OR, 1.439; 95% CI, 1.255–1.65; *p* = 1.9e−7) and “Cardiac arrest” (OR, 1.476; 95% CI, 1.05–2.076; *p* = 0.025). Detailed information about the associations between VAT mass and CVD outcomes is available in Supplementary Table S3.

### Potential Mechanism Identification and Network Construction

To explore the potential biological mechanism of the role of VAT to CVDs, we excavated publicly available summarized data for genetic associations with 90 cardiovascular-related circulating proteins from the Systematic and Combined Analysis of Olink Proteins Consortium (Folkersen et al., 2020). Ninety CVD-related circulating proteins were used as either outcomes or exposures in our network MR analysis.

**FIGURE 4:**
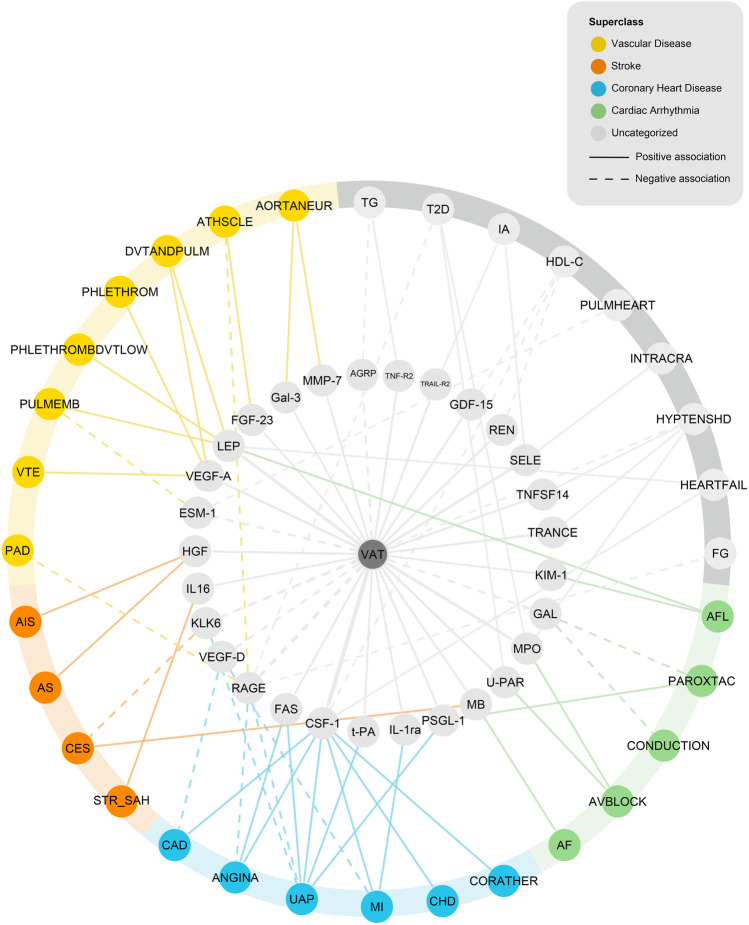
Network analysis of VAT-proteins-CVDs. AGRP, Agouti-related protein; TNF-R2, Tumor necrosis factor receptor 2; TRAIL-R2, TNF-related apoptosis-inducing ligand receptor 2; GDF-15, Growth/differentiation factor 15; REN, Renin; SELE, E-selectin; TNFSF14, Tumor necrosis factor ligand superfamily member 14; TRANCE, TNF-related activation-induced cytokine; KIM-1, Kidney injury molecule 1; GAL, Galanin peptides; MPO, Myeloperoxidase; U-PAR, Urokinase plasminogen activator surface receptor; MB, Myoglobin; PSGL-1, P-selectin glycoprotein ligand 1; IL-1ra, Interleukin-1 receptor antagonist protein; t-PA, Tissue-type plasminogen activator; CSF-1, Macrophage colony-stimulating factor 1; FAS, Tumor necrosis factor receptor superfamily member 6; RAGE, Receptor for advanced glycosylation end products; VEGF-D, Vascular endothelial growth factor D; KLK6, Kallikrein-6; IL16, Pro-interleukin-16; HGF, Hepatocyte growth factor; ESM-1, Endothelial cell-specific molecule 1; VEGF-A, Vascular endothelial growth factor A; LEP, Leptin; FGF-23, Fibroblast growth factor 23; Gal-3, Galectin-3; MMP-7, Matrix metalloproteinase-7; TG, Triglycerides; T2D, Type 2 diabetes; IA, Intracranial aneurysm; HDL-C, High-density lipoprotein cholesterol; PULMHEART, Pulmonary heart disease, diseases of pulmonary circulation; INTRACRA, Nontraumatic intracranial haemmorrhage; HYPTENSHD, Hypertensive Heart Disease; HEARTFAIL, All-cause Heart Failure; FG, fasting glucose; AFL, atrial fibrillation and flutter; PAROXTAC, Paroxysmal tachycardia; CONDUCTION, Conduction disorders; AVBLOCK, AV-block; AF, Atrial fibrillation; CORATHER, Coronary atherosclerosis; CHD, Major coronary heart disease event; MI, myocardial infarction; UAP, Unstable angina pectoris; ANGINA, Angina pectoris; CAD, coronary artery disease; STR_SAH, Stroke, including SAH; CES, Cardioembolic stroke; AS, Any stroke; AIS, Ischemic stroke; PAD, Peripheral artery disease; VTE, Venous thromboembolism; PULMEMB, Pulmonary embolism; PHLETHROMBDVTLOW, DVT of lower extremities; PHLETHROM, Phlebitis and thrombophlebitis (not including DVT); DVTANDPULM, DVT of lower extremities and pulmonary embolism; ATHSCLE, Atherosclerosis, excluding cerebral, coronary and PAD; AORTANEUR, Aortic aneurysm.

Statistically, 50 of 90 tested proteins were affected by VAT (Supplementary Table S4), 85 of 90 proteins were affected by at least one CVD outcome (five proteins were not involved due to the lack of effective instrument variable) (Supplementary Table S5). There was consistency in the direction of outcomes between the genetic associations of VAT with proteins, proteins with CVDs, and VAT with CVDs. A network MR analysis was conducted to combine all directionally consistent results. Finally, 60 VAT-proteins-CVDs axes were identified, including 29 proteins and 32 CVD outcomes (Figure 4; Supplementary Table S6).

Of 29 proteins in the network, a genetically estimated increase of VAT mass was positively associated with 23 proteins, including interleukin and interleukin receptor (IL-1ra, IL16); tumor necrosis factor and tumor necrosis factor receptor (TRANCE, TNFSF14, TNF-R2, TRAIL-R2, FAS); growth factor (VEGF-A, FGF-23, GDF-15, HGF, CSF-1); enzyme (MMP-7, MPO, REN); selectin (PSGL-1, SELE); and others (Gal-3, myoglobin, U-PAR, t-PA, KIM-1, LEP). A genetically estimated increase of VAT mass was negatively associated with six proteins (GAL, ESM-1, RAGE, VEGF-D, KLK6, and AGRP).

The 32 CVD outcomes in the network consisted of four metabolic risk factors (fasting glucose, T2D, HDL-C, TG) and 28 CVD endpoints (due to different database origin, some of them might have overlap), which can be classified into five main categories: coronary heart disease (including diseases of coronary artery and ischemic heart diseases), stroke, cardiac arrhythmia, vascular diseases (including diseases of arteries or arterioles and diseases of veins) and others. Coronary heart disease includes “Coronary artery disease,” “Angina pectoris,” “Major coronary heart disease event,” “Coronary atherosclerosis,” “Unstable angina pectoris,” and “Myocardial infarction”. Stroke includes “Ischemic stroke,” “Any stroke,” “Cardioembolic stroke,” and “Stroke, including SAH.” Cardiac arrhythmia includes “Atrial fibrillation,” “Atrial fibrillation and flutter,” “AV-block,” “Conduction disorders,” and “Paroxysmal tachycardia.” Vascular diseases include 4 diseases of arteries or arterioles (“Aortic aneurysm,” “Atherosclerosis, excluding cerebral, coronary and PAD,” “Pulmonary embolism,” and “Peripheral artery disease”), 4 diseases of veins [“DVT of lower extremities and pulmonary embolism,” “Phlebitis and thrombophlebitis (not including DVT),” “DVT of lower extremities,” and “Venous thromboembolism”].

## Discussion

Obesity is defined by excessive body fat and is often estimated by BMI or body surface area (BSA) based on weight and height calculation. The value of body mass index and VAT mass are strongly correlated at the population level. Given the heterogeneity of obesity, however, individuals with the same BMI could have distinct body fat distribution and metabolic profiles (Powell-Wiley et al., 2021). Therefore, beyond BMI, it is necessary to quantify different body fat depots to evaluate the metabolic status. The visceral adiposity is the most visible marker of ectopic fat deposition and disturbed hormonal milieu (Després, 2012). Anthropometric measures used to estimate VAT includes the measurement of BMI, waist circumference (WC), waist-to-hip ratio (WHR), and waist-to-height ratio (WHtR). Anthropometric indices are available, but insufficiently reflect actual body fat distribution compared with imaging modalities. Imaging techniques used to estimate VAT, including ultrasonography, CT, magnetic resonance imaging (MRI), positron emission tomography (PET)-CT, and PET-MRI, could depict both quantitative and qualitative features of adipose tissue composition (Oikonomou and Antoniades, 2019). Notably, approaches used to quantify VAT may vary in different populations. In participants of Framingham Heart Study, anthropometric measures, including WC and BMI, captured VAT-associated cardiometabolic risk in men but not in women. In women, abdominal CT–based VAT measures more precisely captured the obesity-associated cardiometabolic risk (Kammerlander et al., 2021).

Studies that quantify the volume or area of VAT by imaging techniques support that excess VAT is an indicator of poor CVD outcomes, independent of BMI. Nevertheless, the relationship between VAT expansion and CVDs is still unclear, as both fewer and more VAT components have been associated with higher risks of CVDs. Two major ideas may account for this phenomenon. The first one is the biological variability of different depots depending on the location and metabolic state. Other than deteriorative effects, VAT components could exert protective effects on the cardiovascular system. Under normal conditions, PVAT had a protective effect on vascular biology by secreting vasorelaxant molecules (Nosalski and Guzik, 2017). Under the pathological condition, the phenotype of PVAT shifted (Gollasch, 2017). Another perspective is that CVDs and other metabolic disorders might affect VAT reversely. For instance, myocardial-derived oxidation products increase the level of adiponectin, a protective protein in regulating metabolism, in the adjacent EAT (Antonopoulos et al., 2016b). In contrast, atrial natriuretic peptide (ANP) secreted by the myocardium contributes to the pro-arrhythmogenic crosstalk between EAT and atrial myocardium (Suffee et al., 2017). Thus, the effect of CVDs on VAT may result in reverse causation bias when determining the effect of VAT on CVDs in observational studies. Besides, observational studies are vulnerable to unmeasured confounding based on available data. A few observational studies have been reported the association between the volume or area of VAT and metabolic risk factors (Fox et al., 2007; Liu et al., 2010; Abraham et al., 2015), hypertension, diabetes (Liu et al., 2010), coronary artery disease (Marques et al., 2010; Ohashi et al., 2010), embolic stroke (Muuronen et al., 2015), and metabolic syndrome (Shah et al., 2014). However, the causal relationship between VAT mass and a wide range of CVD outcomes is still unclear, and the potential mechanism is unknown. Most experimental studies about the relationship between VAT and CVDs focus on the impact of specific depots of VAT on CVDs, like EAT (Wong et al., 2017; Packer, 2018; Ernault et al., 2021) PAT (Al Chekakie et al., 2010; Thanassoulis et al., 2010), and PVAT (Xia and Li, 2017), but not the impact of overall VAT.

To address these issues, we conducted the most comprehensive MR analysis to determine the causality between VAT and CVDs. Consistent with previous observational researches, we confirmed detrimental impacts of VAT on cardiometabolic traits including lipid profiles and glucose profiles (Fox et al., 2007; Liu et al., 2010). We confirmed the adverse effects of VAT accumulation on CAD (Marques et al., 2010; Ohashi et al., 2010; Bachar et al., 2012), vascular disease (Lim and Meigs, 2014; Farb and Gokce, 2015), stroke (Muuronen et al., 2015), and AF (Al Chekakie et al., 2010; Thanassoulis et al., 2010; Shin et al., 2011; Mazurek et al., 2014). Obesity-induced vascular dysfunction could explain the causality of VAT with coronary artery disease and other vascular diseases (Farb and Gokce, 2015). In terms of stroke and its subtypes, the effect of VAT expansion might be partially due to hemodynamic derangements as observed in obese subjects (De Pergola and Pannacciulli, 2002; Darvall et al., 2007). The present study first showed that VAT was the risk factor for atrioventricular-block, conduction disorders, and paroxysmal tachycardia. In summary, our findings demonstrated the extensive deleterious effects of VAT on CVDs. Notably, we did not detect the significant association between VAT mass and systolic/diastolic blood pressure except using the contamination mixture method, which does not align with the evidence that the excess VAT was positively associated with hypertension (Ishikawa et al., 2010; DeMarco et al., 2014; Hall et al., 2015; Lorbeer et al., 2018). Additionally, we found that the genetically determined VAT mass was negatively linked with DBP in the MR-Egger method. This inconsistent result may be attributed to the unbalanced horizontal pleiotropy in the estimates of VAT mass to DBP (P_Egger intercept_ = .002).

It is well-documented that the visceral fat, compared with the subcutaneous compartment, is much more metabolically active (Ibrahim, 2010; Alexopoulos et al., 2014). VAT, as an endocrine organ, secrets plenty of adipokines into circulation which contributes to the systematic control of metabolic homeostasis, inflammatory response, and various functions. To explore the mediate mechanism underlying the association between VAT and CVDs, we introduced 90 circulating proteins from 15 cohorts as potential mediators (Folkersen et al., 2020). Most of the proteins are prognostic biomarkers or drug targets of CVDs. After a strict screening process, we reserved the proteins participating in the adverse effect of VAT on CVDs. We constructed a network involving 29 proteins, 32 CVD outcomes, and 60 pathways. Most VAT-associated proteins increased the incidence of CVDs, and they could be detected in adipose tissue secretome based on the previous study (Hauner, 2005; Dahlman et al., 2012; Lehr et al., 2012; Uhlén et al., 2019). We hence hypothesized that VAT could secret these proteins as circulating adipokines, and then affect the homeostasis of the circulation system. As for the proteins that decreased the incidence of CVDs, VAT expansion could reduce the expressions of which. The underlying mechanism needs further researches. In summary, our profiling provides a comprehensive understanding of linking VAT with the pathogenesis of CVD via these circulating proteins. Most of the proteins in the network were involved in energy metabolism (MB, VEGF-D, CSF-1, RAGE, LEP, FASL, MPO, GAL, VEGF-A, PSGL-1, MMP-7, TNF14, TNF-R2, t-PA, AGRP, REN, GDF-15, and TRAIL-R2), inflammation (HGF, VEGF-D, KLK6, RAGE, LEP, FASL, MPO, VEGF-A, SELE, PSGL-1, ESM-1, TNF14, IL-1ra, IL16, TNF-R2, and REN), or angiogenesis (HGF, VEGF-D, LEP, MPO, VEGF-A, SELE, PSGL-1, and ESM-1).

Among these proteins, genetically estimated VAT mass had the highest OR value for the expression of leptin. The OR per 1-SD increase of VAT mass was 1.742 (95% CI, 1.607–1.888) for the level of leptin. Leptin is one of the most extensively investigated adipokines with an adverse association with CVDs (Sweeney, 2010). Leptin plays a crucial role in metabolism, apoptosis, extracellular matrix remodeling, endothelial dysfunction, and thrombosis. In our network, excessive VAT increased the level of leptin and further increased the incidence of “all-cause heart failure,” “atrial fibrillation and flutter,” “DVT of lower extremities,” “pulmonary embolism,” and “DVT of lower extremities and pulmonary embolism.” The effect of leptin on extracellular matrix remodeling and cardiac hypertrophy might contribute to the development of HF (Sweeney, 2010). The effect of leptin on endothelial dysfunction might contribute to DVT of lower extremities and pulmonary embolism. Limited studies have reported the effect of leptin on incident AF and provided a discrepant conclusion. An observational study showed that patients with AF had higher serum leptin levels compared with controls (Anaszewicz et al., 2019). Two animal experiments have noted that leptin signaling is essential for developing atrial fibrosis and AF evoked by angiotensinII (Fukui et al., 2013) or high-fat diet (Fukui et al., 2017), while another study reported that leptin attenuates isoproterenol-induced arrhythmogenesis (Lin et al., 2013). The different inducers of the AF model may underlie this discrepancy. Future study to determine the effect of leptin secreted by visceral adiposity on AF is expected.

## Strengths and Limitations

Our research has multiple strengths. Firstly, we conducted the most comprehensive analysis of the causality between VAT and CVDs. We confirmed the effect of excess VAT on a wide range of cardiometabolic risk factors and CVD endpoints. Our finding indicated that VAT accumulation exacerbated cardiometabolic profiles extensively. Secondly, our MR design reduced confounding and reverse causation bias compared with the observational study. A large sample size of GWAS summary statistics provided a substantial statistical performance to examine the association between VAT and CVD outcomes. Thirdly, we involved cardiovascular-related circulating proteins as potential mediators between VAT and CVDs and conducted a network analysis.

Some limitations need to be noted. Firstly, the results from an MR study can be violated by pleiotropy, which describes a genetic variant associated with multiple traits. However, in most cases, sensitivity analyses using MR-Egger, weighted median, and contamination mixture method provided less precise estimates but consistent direction. Secondly, most participants of the GWASs were of European ancestry, which reduced the generalizability of the conclusion. Thirdly, it should be noted that the function and deposition of adipose tissue differ by sex. Males tend to have more visceral fat that is highly correlated to increased cardiovascular risk; whereas females tend to have more subcutaneous fat (Palmer and Clegg, 2015). It has been reported that the positive associations between VAT and CVD outcomes were stronger in females compared with males (Karlsson et al., 2019; Kammerlander et al., 2021). However, we did not have the GWAS data for solely predicting VAT mass in each gender, so we cannot determine whether the associations between VAT and CVDs vary in gender. Finally, we only included 90 CVD proteins, as they represent the most promising targets. Further studies might include more proteins from large-scale pQTL studies.

### Prospective Future

Observational studies demonstrated expanded visceral adiposity as a CVD risk factor. Our MR results added to this body of evidence and presented the causal link between VAT mass and CVDs. However, not only the quantity of VAT but also the quality of VAT is important. Future studies are expected to evaluate the associations between different types of VAT (white adipose tissue and brown adipose tissue) and CVDs. Besides, the external validation and confirmation of our findings in populations of different genders, different races, and different ages may be necessary. The proteins in network analysis provided diagnostic or predictive tools and could be used as therapeutic targets in individuals with excess VAT. Reducing the quantity of VAT (Verheggen et al., 2016; Rao et al., 2019) and targeting the adipokines secreted by VAT might help to prevent or treat CVDs. Future randomized trials are expected to investigate the efficiency.

## Conclusion

Mendelian randomization analysis showed that VAT mass was associated with a wide range of CVD outcomes including coronary heart disease, cardiac arrhythmia, vascular diseases, and stroke. A few circulating proteins may be the mediators between VAT and CVDs. Future studies are warranted to validate these findings.

## Data Availability

The original contributions presented in the study are included in the article/Supplementary Material, further inquiries can be directed to the corresponding authors.
